# Comparison of Single-Use Negative-Pressure Wound Therapy (sNPWT) and Standard Dressings Applied to the Same Patient During Bilateral Tissue Expander-to-Implant Exchanges

**DOI:** 10.3390/cancers17010003

**Published:** 2024-12-24

**Authors:** Maja Molska, Magdalena Wojciech, Dawid Murawa

**Affiliations:** 1 Clinical Department of General and Oncological Surgery, University Hospital in Zielona Góra, 65-046 Zielona Góra, Poland; 2 Department of Surgery and Oncology, Collegium Medicum, University of Zielona Góra, 65-046 Zielona Góra, Poland; 3 Institute of Mathematics, University of Zielona Góra, 65-417 Zielona Góra, Poland

**Keywords:** breast cancer, breast reconstruction, wound healing, sNPWT, scar

## Abstract

Background: Breast cancer is the most common cancer among women. The number of cases is increasing among young women, and consequently, breast reconstructions are performed more often. Postoperative complications, wound healing, and the quality of scars influence the final cosmetic outcomes. The aim of the study was to investigate the effect of using a negative-pressure therapy compared to a standard dressing on two postoperative wounds in the same woman, after bilateral tissue expander replacement surgery—when identical healing conditions occur. Methods: This study includes a single-center evaluation of the application of two different dressings during bilateral exchanges of tissue expanders with breast implants. The study included 22 patients with a history of breast cancer after bilateral mastectomy with two-stage reconstruction. During the second stage, a sNPWT was applied to one breast and a standard dressing to the other. Results: A statistically significant difference in skin elasticity in favor of sNPWT was visible after 7 days, while the greatest difference was visible in the evaluation after 6 months (mean of 0.806 vs. 0.607). A difference in temperature measurements was shown 7 days after the procedure. However, after 30 days, these measurements were similar. There was also a reduced incidence of postoperative complications as well as seroma accumulation in the breasts with negative pressure. Conclusions: The results indicate faster healing, better scar quality, and improved skin elasticity in breasts with sNPWT compared to the standard dressing. The number of postoperative complications were also reduced, which is especially important in high-risk patients.

## Simple Summary

This article presents the results of using two different dressings (sNPWT and SD) in the same woman after bilateral exchanges of tissue expanders for breast implants. It compares the wound healing under identical conditions and time. The study included 22 women who had a standard dressing applied to one breast after the procedure and a negative-pressure wound therapy to the other. This allows for a precise comparison of the influence of dressings on the method and speed of wound healing, occurrence of postoperative complications, and scar quality and elasticity in the long term.

## 1. Introduction

Breast cancer is the most common fatal disease among women [1]. Population awareness is increasing, and more young patients are being diagnosed with cancer [2,3]; therefore, the number of breast reconstructions is also growing significantly. There is a noticeable trend that patients decide to undergo mastectomy despite being qualified for breast-conserving treatment [4,5], and contralateral risk-reducing mastectomies are being performed more often too [6,7]. This is mainly due to the fear and anxiety about future outcomes.

The postoperative period, wound healing, and the appearance of scars directly affect the patient’s quality of life. Losing an implant is one of the worst and most unpleasant complications that both women and surgeons are afraid of [8]. Many factors influence wound healing and the risk of postoperative complications, such as high BMI, smoking, or previous radiotherapy [9].

Fortunately, we know many ways to deal with complications and, importantly, we have tools to prevent possible complications. One of them is single-use negative-pressure wound therapy (sNPWT). This therapy uses a small, lightweight, portable suction device connected to an absorbent, adhesive dressing. Its purpose is, among other things, to increase local blood flow, reduce tension across surgical incisions, protect against contamination, and therefore reduce the risk of surgical site infections (SSIs), wound dehiscence, or keloid formation [10,11,12].

Many studies have been conducted on negative-pressure therapy [13,14,15,16,17]. However, there is still little talk about its use in breast surgery and its effect on the quality and elasticity of the scar. This seems to be particularly important in the case of repeated surgery, such as in two-stage breast reconstruction. Re-incised scar tissue heals worse and is more prone to the formation of keloids. Importantly, the tendencies for good or bad wound healing are individualized; hence, we must also consider independent variabilities to compare the dressings [18,19].

In our research, we wanted to investigate the effect of using a negative-pressure dressing compared to a standard dressing on two postoperative wounds in the same woman after bilateral tissue expander replacement surgery with breast implants in identical healing and demographic conditions. We studied the influence of different dressings on the postoperative period and wound healing.

## 2. Materials and Methods

**Figure 1:**
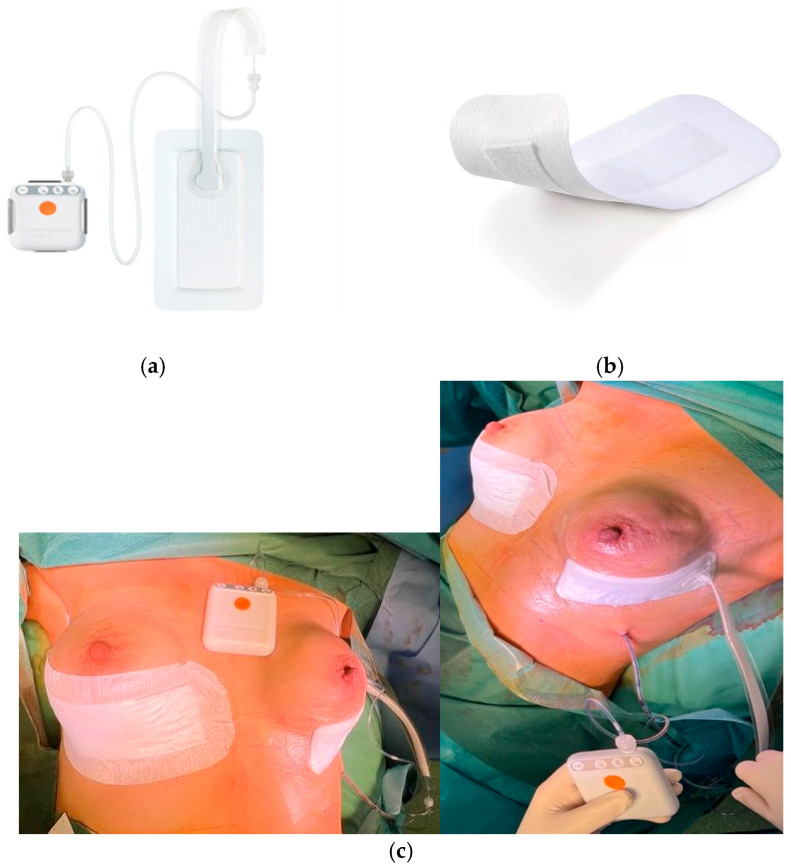
Photos of dressings ((**a**) PICO7^®^, (**b**) Cosmopor^®^, (**c**) dressing application method).

This single-center prospective study aimed to evaluate the application of two different dressings in the same patient during bilateral exchanges of tissue expanders with breast implants. The study included adult patients with a history of breast cancer after bilateral mastectomy with two-stage reconstruction. During the second stage of reconstruction, a single-use negative-pressure therapy was applied to the sutured wound of one breast, and a standard dressing was applied to the other. We used Smith & Nephew PICO7^®^ dressings, the devices we used in our center in everyday practice, and standard sterile dressings for postoperative wounds—Cosmopor^®^ by Hartmann (Figure 1).

PICO7^®^ is a small device with an electric motor-driven vacuum pump connected to an absorbent dressing. It is used for difficult-to-heal and postoperative wounds in high-risk patients. It creates a negative pressure of −80 mmHg and absorbs up to 300 mL of exudate, keeping it away from the wound. This dressing is left on the wound for 7 days without changing. The device increases local blood flow, stimulates the immune system response, reduces tissue tension, and improves the lymphatic system’s functioning. Of all the sNPWTs used in our center, PICO7 seems to be the most convenient for both physicians and patients. It adapts well to the wound and works correctly and quietly. In addition, the amount of research conducted on these devices makes them proven and reliable [20,21,22,23].

Cosmopor^®^ is an adhesive dressing designed for postoperative wounds. It is also used for sterile dressing of minor cuts, e.g., as part of first aid. It is a typical dressing with a small absorbent layer, applied in the postoperative period when the wound requires standard protection.

Each surgery was performed by the same team in the same conditions, maintaining the sterility of equipment and the operating room. We applied the dressings in the operating room immediately after the procedure. We kept the sNPWT application on the wound for 7 days, and changed the standard dressings approximately every 2–3 days. We assessed the difference in wound healing, the occurrence of postoperative complications, skin elasticity, and scar quality, the temperature around the wound, and the patients’ perceptions. Check-ups were carried out a week after the surgery—when we removed the dressings—and a month and half a year after. The elasticity of the skin and its temperature were measured objectively using a specialized device.

**Figure 2:**
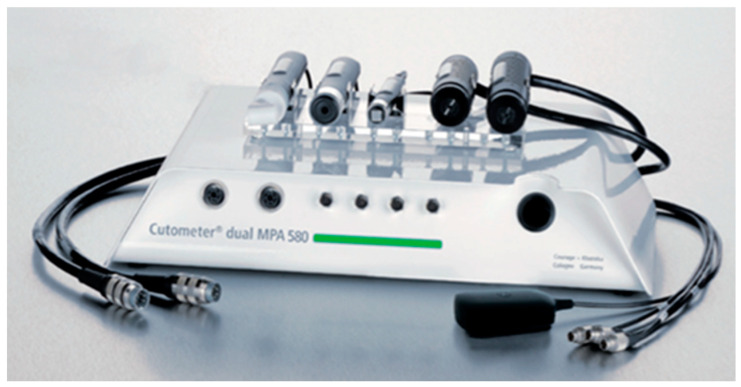
Cutometer.

We used a Cutometer probe with a 6 mm round entrance diameter connected to the Cutometer^®^ dual MPA 580 Plus (Figure 2). The device generates a negative pressure (up to 450 mbar) which sucks the skin through an opening in the probe and measures its vertical deformation and ability to return to its original shape. Skin property studies using suction force are widely used worldwide. The Cutometer^®^ is the most commonly used piece of medical equipment for objectively assessing scars [24,25]. As a result of the negative pressure generated by the probe and applied to the skin surface, its superficial layers are deformed and lifted. The height of the lift varies depending on the skin condition, including the connective tissue structures in the epidermis and dermis.

Skin surface temperature was measured using infrared radiation by a Skin-Thermometer ST 500 probe connected to the same Cutometer^®^. The temperature was measured without contact, i.e., without applying any force to the skin, so as not to additionally affect the skin microcirculation in the selected area.

Elasticity was measured the day before the procedure—at the place of the scar from the previous procedure—a week later, and during the check-up after six months. The temperature of the surgical area was measured a week after the procedure—after removing the dressings—and during the check-up after a month.

Patients’ characteristics were also taken into account (Table 1). If the patient had radiotherapy to either breast or the elasticity of the scars was significantly different, the negative-pressure dressing was always placed on the breast at higher risk of postoperative complications.

## 3. Results

We performed 22 bilateral expander exchanges with breast implants from April 2022 to June 2023 with a six-month follow-up. We applied 22 standard and 22 sNPWT dressings.

**Figure 3:**
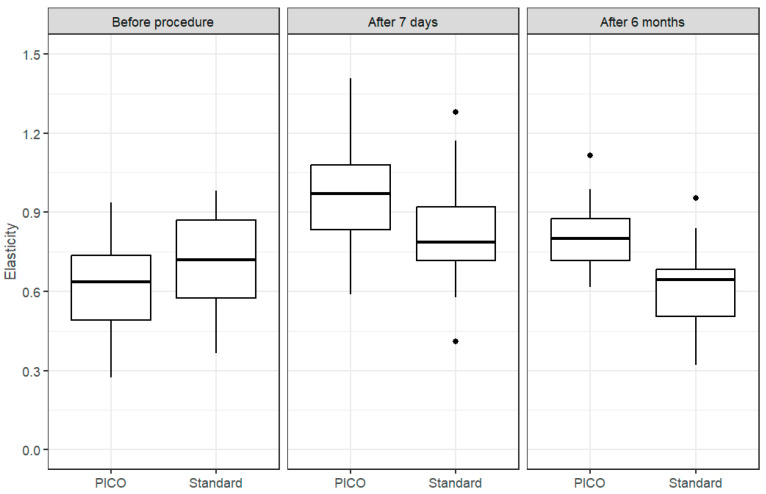
Skin elasticity measurement for patients treated with PICO and standard dressings: before the procedure, after 7 days, and after 6 months.

Figure 3 shows the distribution of skin elasticity values for both types of dressings. In case of elasticity measurements, as mentioned in the methodology, the PICO dressing was applied to the skin with worse parameters, i.e., the mean value was 0.618 vs. 0.702 and median 0.637 vs. 0.72. In measurements after 7 days, an opposite trend was observed: mean 0.954 vs. 0.827 and median 0.971 vs. 0.788. These were new wounds; hence, the elasticity was better than in the measurements on the scars before the procedure. However, a significant difference in favor of negative-pressure therapy is visible in the final measurements after 6 months: mean 0.806 vs. 0.607 and median 0.8 vs. 0.644, when the surgical incisions were already scarred.

**Figure 4:**
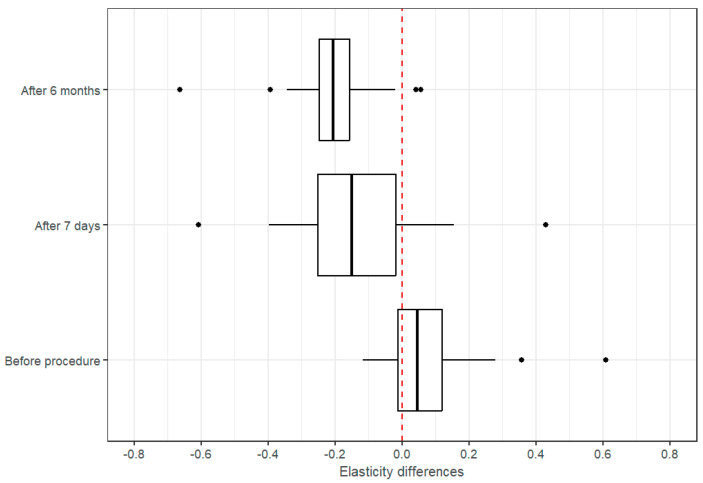
Elasticity differences at the surgical site with the standard dressing and PICO. The red dashed line shows the difference value indicating equal elasticity for both dressings.

Differences in elasticity in the surgical site were determined using the standard and PICO dressings (Figure 4). The significance of these differences was verified using the paired parametric *t*-test or, in the case of failure to meet its assumptions, the nonparametric Wilcoxon test for paired samples. In the case of skin elasticity measurements before the procedure, statistically significant results were obtained, indicating a higher mean skin elasticity in the group with the standard dressing (*p*-value = 0.011). The differences in skin elasticity measurements 7 days and 6 months after the procedure were also statistically significant. However, the direction of change was the opposite, i.e., skin elasticity was higher with the PICO dressing compared to the standard one (*p*-value = 0.007 and *p*-value < 0.001, respectively).

As can be noticed, initially, the difference in elasticity indicated better skin quality in patients who had standard dressings applied. However, this changed over time to the advantage of sNPWT, where the greatest difference is visible in measurements after 6 months.

**Figure 5:**
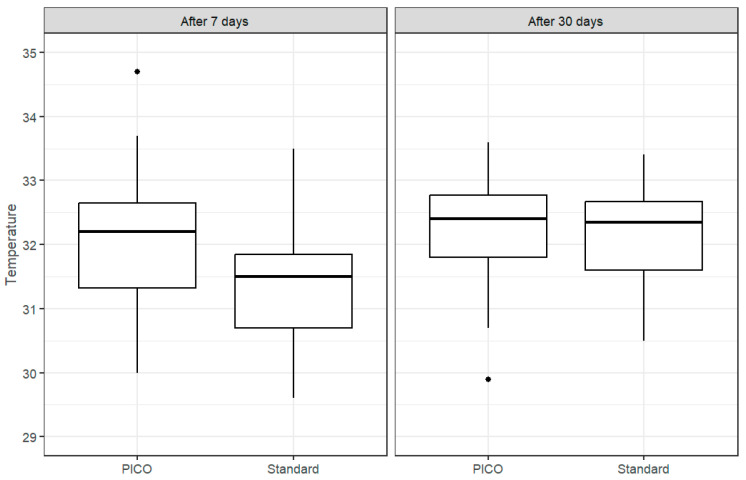
Temperature measurements for patients treated with PICO and standard dressings: after 7 days and after 30 days.

**Figure 6:**
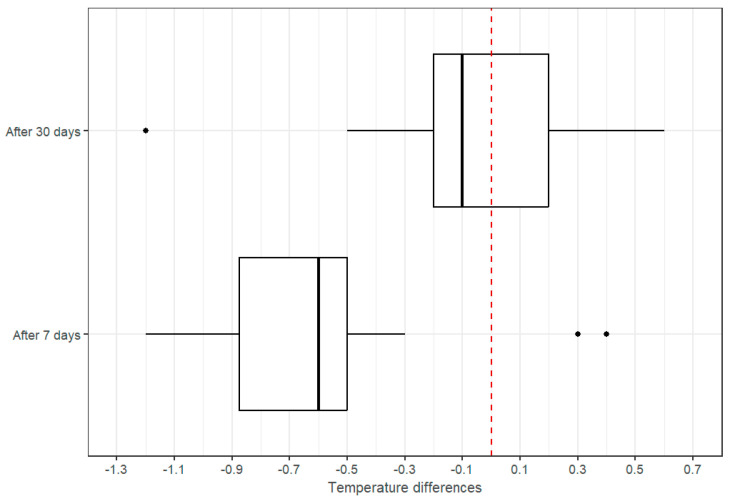
Temperature differences at the surgical site with the standard dressing and PICO. The red dashed line shows the difference value indicating equal temperature for both dressings.

The temperature of the surgical site was measured 7 days after the procedure, after the dressings were removed, and on the 30th day after the procedure (Figure 5 and Figure 6). It was shown that the temperature one week after the procedure was statistically significantly different between the breasts and was higher in the PICO site (*p*-value < 0.001). The mean temperature was 32.0 vs. 31.4 °C, respectively, and the median was 32.2 vs. 31.5 °C. This is probably due to increased blood flow and faster tissue regeneration. However, we did not detect a statistically significant difference in the temperature measurements one month after the procedure (*p*-value = 0.515). The mean and median for both dressings were approximately equal and were 32.2 and 32.4 °C, respectively.

Therefore, researchers conclude that the temperature difference after 7 days indicates a higher temperature after sNPWT application, whereas after 30 days, the difference did not show a statistically significant value.

In the immediate postoperative period, what was particularly interesting to researchers was that patients reported significantly less pain on the side with the vacuum dressing. This was not related to performing a different surgical technique or individual variables.

In postoperative check-ups, we noticed significantly less seroma accumulation in the reconstructed breast with PICO compared to the breast with standard dressing. In the weekly check-up, it was on average 60 vs. 20 mL, and after a month, it was 40 vs. 0 mL. We had two more serious complications—both occurring on the side of the standard dressing. In one patient, worse and longer wound healing was experienced, while in the other patient, a skin fistula developed, which required removal of the implant. The second woman was burdened with diabetes and nicotine addiction. No major complications appeared in the breasts with the PICO.

**Figure 7:**
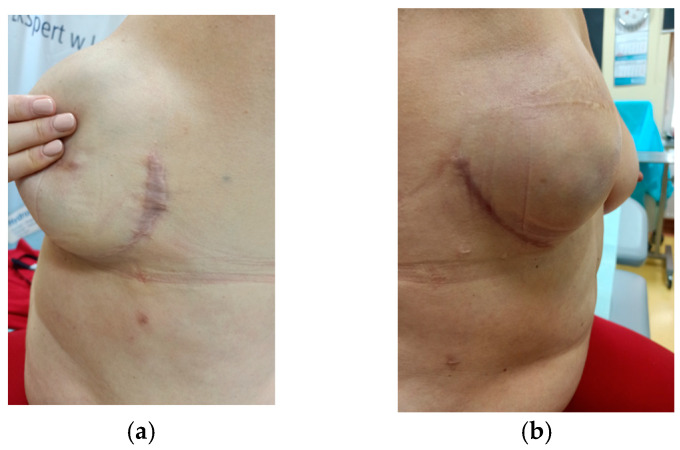
Patient No. 1 ((**a**) standard dressing; (**b**) PICO7).

**Figure 8:**
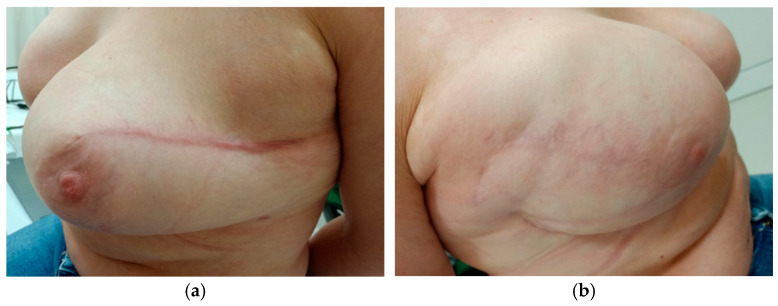
Patient No. 2 ((**a**) standard dressing; (**b**) PICO7).

In the six-month follow-up, we assessed not only the elasticity but also the visual quality of the scars. It was noticeable that the scars after application of negative pressure were paler, less conspicuous, and less prone to the formation of keloids. The comparison can be seen in photos—Figure 7 and Figure 8.

The photographs of these two patients vividly illustrate the typical differences observed between the use of the dressings. The authors evaluated the visual quality of the scars by examining their color, width, and the presence of keloid formation. These images provide compelling evidence of the impact of sNPWT on enhancing scar quality.

## 4. Discussion

Breast reconstruction is a unique surgical procedure that provides patients undergoing mastectomy not only with the most important element—treatment of breast cancer and oncological safety—but also with significant psychosocial and aesthetic benefits. Currently, breast oncological surgery has become a field closely related to plastic surgery. Contralateral, risk-reducing, or prophylactic mastectomies are becoming more common, associated with the increasing number of genetic tests and detected mutations. Patient expectations are also growing. Therefore, especially in the high-risk group, it would be worthwhile to apply preventive measures to minimize the number of postoperative complications, better control wound healing, and improve the quality of scars.

An extensive literature review was presented by Saunders et al. [26] This study aimed to determine whether prophylactic use of a sNPWT device reduced the incidence of surgical complications after closed surgical incisions compared with conventional dressings. The researchers included 29 studies with a total of 5614 patients. Various types of procedures were compared, including colorectal, orthopedic, and breast surgeries. The results were clear. Overall, the odds of SSI were reduced by 63% with sNPWT. For other complications, negative-pressure wound therapy also showed benefits, including wound dehiscence, reduced seroma, and necrosis. It was also noticeable that the length of hospital stay was shorter in patients receiving sNPWT.

An interesting meta-analysis was conducted by Mrad et al. [27]. The purpose of the research was to identify potential risk factors associated with postoperative complications after breast reconstructions. They included 33 studies, and among them, over 100,000 patients. The overall population included patients undergoing breast reconstructions following mastectomy. Techniques primarily involved implants, tissue expanders, and autologous reconstructions. This analysis shows that risk factors like age, diabetes, smoking history, high blood pressure, and body mass index (BMI) have a crucial effect on multiple complications after breast reconstructions. Similar risk factors are discussed by Roubaud et al. in their article [28], also taking into account the risk of complications in large, ptotic breasts. Thuman et al., on the other hand, focused on the problems resulting from previous radiotherapy [29]. Another important risk factor, as Cassela et al. pointed out [30], is the qualification of patients for pre- or subpectoral breast reconstruction, and direct-to-implant or two-stage reconstruction. Decision-making is not clearly standardized, and there is a risk of wound complications, partial ischemia, or tissue necrosis in the case of inadequate assessment.

Surgeons must be aware of these predictors before performing breast reconstructions on their patients to protect them and minimize the risk of postoperative complications. One of the very effective and helpful tools for this is sNPWT. As we can read in Irwin’s article [31], where the researchers compared sNPWT (126 cases) with standard dressings (181 cases) after prepectoral implant breast reconstructions, they noted a significant reduction in postoperative complications with the use of NPWT. It reduces the rate of wound dehiscence and implant loss. In addition to the clinical benefits, this approach has also been shown to be cost-saving and shorten hospital stays compared with standard dressings.

Similar conclusions were presented in Wareham’s article [32]. Importantly, in this study, the NPWT cohort had higher rates of macromastia symptoms, BMI, and ASA, which put these patients in the high-risk group. Therefore, the authors suggest that NPWT should be considered in oncoplastic patients, especially those at increased risk of postoperative complications.

Our study also showed that sNPWT significantly reduces the incidence of postoperative complications. It seems relevant that we used two different dressings in the same woman, which allowed us to compare the healing outcomes under identical conditions. We applied negative-pressure dressings to the breasts, which were initially in worse condition, and the final results still favored sNPWT. We were also interested in the fact that patients reported less pain in the immediate postoperative period on the side with sNPWT. This may have been due to increased blood flow and reduced occurrence of swelling. Of course, our group is relatively small; however, it clearly shows that the use of sNPWT not only provides clinical benefits in the short postoperative period but also makes scars paler and more delicate, and reduces the risk of keloid formation, which is also important for the good quality of life of patients treated for breast cancer in the present day.

Obviously, not every patient after breast reconstruction requires sNPWT; however, if significant risk factors are present, such as those described above, it seems highly reasonable to consider the use of sNPWT, to avoid complications, including the worst—loss of reconstruction.

## 5. Conclusions

The results indicate faster healing of postoperative wounds, better scar quality, and improved skin elasticity in breasts treated with single-use negative-pressure therapy compared to the standard dressing. The number of postoperative complications is also reduced, which is especially important in high-risk patients. Vacuum dressings can be successfully used to prevent postoperative failures, including implant loss, which is one of the worst complications and significantly deteriorates patients’ quality of life. Further research on this topic is necessary.

## Figures and Tables

**Table 1:** Patients’ characteristics.

Characteristic	Number	
Women	22	
Breast cancer	22	
Bilateral exchanges	22	
Age (mean)	45	
BMI (mean)	23	
Comorbidities	yes: 9 (40.9%)	no: 13 (59.1%)
Smoking	yes: 2 (9.1%)	no: 20 (90.9%)
Chemotherapy	yes: 19 (86.4%)	no: 3 (13.6%)
Radiotherapy	yes: 5 (22.7%)	no: 17 (77.3%)
Hormone therapy	yes: 12 (54.5%)	no: 10 (45.5%)

## Data Availability

The research data can be available after direct contact with the principal investigator—majaa.molska@gmail.com.
